# Self–Supporting Mn–RuO_2_ Nanoarrays for Stable Oxygen Evolution Reaction in Acid

**DOI:** 10.3390/molecules28237727

**Published:** 2023-11-23

**Authors:** Mengting Deng, Yulong Tang, Zhiyi Lu, Yunan Wang, Yichao Lin

**Affiliations:** 1 School of Materials Science & Chemical Engineering, Ningbo University, Ningbo 315211, China; dengmengting@nimte.ac.cn (M.D.);; 2 Key Laboratory of Advanced Fuel Cells and Electrolyzers Technology of Zhejiang Province, Ningbo Institute of Materials Technology and Engineering, Chinese Academy of Sciences, Ningbo 315201, China; 3 School of Chemical Science, University of Chinese Academy of Sciences, Beijing 100049, China

**Keywords:** ruthenium oxide, water electrolysis, oxygen evolution reaction, proton exchange membrane

## Abstract

Currently, the process of an acidic oxygen evolution reaction (OER) necessitates the use of Iridium dioxygen (IrO_2_), which is both expensive and incredibly scarce on Earth. Ruthenium dioxygen (RuO_2_) offers high activity for acidic OERs and presents a potential substitution for IrO_2_. Nevertheless, its practical application is hindered by its relatively poor stability. In this study, we have developed Mn–doped RuO_2_ (Mn–RuO_2_) nanoarrays that are anchored on a titanium (Ti) mesh utilizing a two–step methodology involving the preparation of MnO_2_ nanoarrays followed by a subsequent Ru exchange and annealing process. By precisely optimizing the annealing temperature, we have managed to attain a remarkably low overpotential of 217 mV at 10 mA cm^−2^ in a 0.5 M H_2_SO_4_ solution. The enhanced catalytic activity of our Mn–RuO_2_ nanoarrays can be attributed to the electronic modification brought about by the high exposure of active sites, Mn dopant, efficient mass transfer, as well as the efficient transfer of electrons between the Ti mesh and the catalyst arrays. Furthermore, these self–supported Mn–RuO_2_ nanoarrays demonstrated excellent long–term stability throughout a chronoamperometry test lasting for 100 h, with no discernible changes observed in the Ru chemical states.

## 1. Introduction

Hydrogen is an adaptable and promising clean energy carrier that has the potential to play a significant role in reducing carbon dioxide emissions. In contrast to the prevalent use of natural gas reforming for hydrogen production in the industry, the electrochemical process of water splitting, empowered by renewable energy sources like solar and wind energy under more moderate conditions, emerges as a considerably more desirable option since it leaves no carbon footprint [1,2,3,4,5,6]. The technique of electrochemical water splitting encompasses two established methods, namely alkaline water electrolysis (AWE) and proton exchange membrane–based water electrolysis (PEMWE) [7,8]. Compared to AWE, PEMWE offers several advantages, including rapid response, low Ohmic loss, high gas purity, and high current density. However, the acidic environment of PEMWE on the anode requires the use of noble iridium dioxygen (IrO_2_) electrocatalysts, which are prohibitively expensive and scarce on Earth. The water electrolysis process involves two reactions: the hydrogen evolution reaction (HER) on the cathode and the oxygen evolution reaction (OER) on the anode [9,10]. It has been observed that the energy barrier of an OER with a four–electron transfer is significantly higher than that of an HER with a two–electron transfer, resulting in a slower, sluggish kinetic process [11,12,13,14]. Consequently, the efficiency of water electrolysis heavily relies on the anodic OER. Hence, there is a pressing need for the development of efficient and affordable electrocatalysts demonstrating high activity and stability.

In contrast to the commercial PEMWE catalyst IrO_2_, ruthenium dioxygen (RuO_2_) exhibits much higher activity for acidic OERs and is more cost effective. Remarkably, Ru is the least expensive noble metal, costing only about one–tenth the price of Ir. However, the stability of RuO_2_ is unsatisfactory due to its greater dissolution in acid compared to Ir [15,16]. Additionally, the electron transfer efficiency is influenced by the contact between the electrocatalysts and the substrate, which notably impacts their activity and stability [17,18]. Unlike powdered catalysts, self–supported catalysts do not require additional binders, conductive agents, or collectors, thereby greatly facilitating charge transfer and electron transfer. Nevertheless, there are only a few instances of self–supported Ru–based nanostructures for acidic OERs [19,20]. For instance, Qu et al. synthesized Ru–NiFe–P nanosheets on 3D self–supported nickel foam [19], which displayed an overpotential of 244 mV at 10 mA cm^−2^. He et al. employed bifunctional metal–organic frameworks (MOFs) to achieve Ru doping, resulting in RuCo–CAT with superior OER activity compared to RuO_2_ [20].

In this study, Mn–doped RuO_2_ (Mn–RuO_2_) nanoarrays on titanium (Ti) mesh are prepared through an ion exchange and annealing process. Ti mesh is chosen as the substrate due to its excellent biocompatibility, superior resistance to acidic corrosion, and conductivity. The incorporation of Mn dopants effectively modifies the electronic structure and enhances the OER activity. Moreover, the nanoarray morphology facilitates mass transfer in the OER process. The resulting Mn–RuO_2_ nanoarrays exhibit outstanding activity and stability for acidic OERs, with a low overpotential of 217 mV at 10 mA cm^−2^, and remain stable for over 100 h.

## 2. Results and Discussions

**Figure 1:**
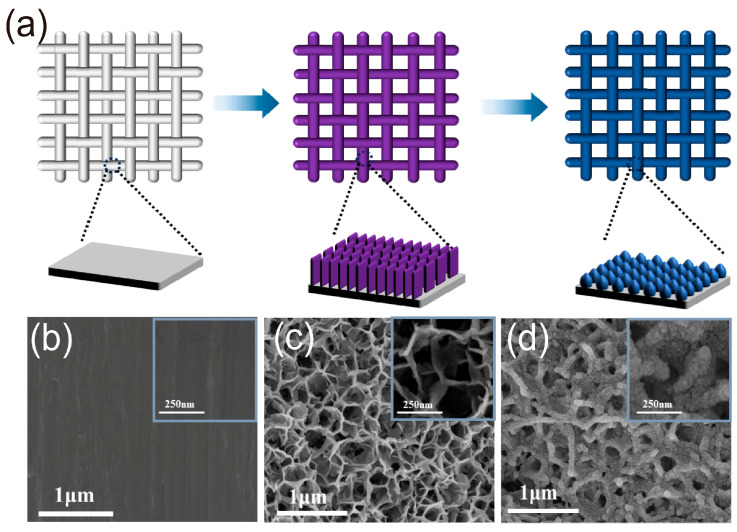
Schematic illustration of preparation of Mn–RuO_2_ nanoarrays (**a**) and SEM images of (**b**) Ti mesh, (**c**) α–MnO_2_ nanoarrays on Ti mesh, and (**d**) Mn–RuO_2_ (300) nanoarrays.

Figure 1a illustrates the preparation of Mn–RuO_2_ nanoarrays using Ti mesh as the substrate by a two–step strategy. In order to obtain morphology features of the Ti mesh, α–MnO_2_ nanoarrays, and Mn–RuO_2_ nanoarrays, scanning electron microscope (SEM) was employed. Figure 1b displays the clean and smooth surface of the Ti mesh. Initially, α–MnO_2_ nanoarrays are hydrothermally grown on the Ti mesh following a previously reported method [21]. A comprehensive description of the synthesis procedure can be found in the materials and methods section. Subsequently, the α–MnO_2_ nanoarrays are immersed in a solution of ruthenium trichloride (RuCl_3_) for the exchange of Ru. The resulting product is then annealed at various temperatures to generate Mn–RuO_2_ nanoarrays, denoted as Mn–RuO_2_ (T, T = 250, 300 or 350 °C). The resulting α–MnO_2_ nanoarrays exhibit a vertically grown nanosheet morphology on the Ti mesh, connected with each other to form minute apertures measuring approximately 0.5 µm in diameter, as depicted in Figure 1c. The Mn–RuO_2_ (T, T = 250, 300 or 350 °C) nanoarrays demonstrate a similar morphology to that of α–MnO_2_ nanoarrays but with a noticeably rougher surface, as evident in Figure 1d, Figure S1, and S2. 

The Ru content on the Ti mesh plays a crucial role in assessing the catalyst for practical application. An excessively high Ru loading becomes redundant and lacks significance due to its relatively high price. To assess the Mn–RuO_2_ nanoarrays loaded on the Ti mesh and determine the Mn/Ru molar ratio, an element analysis is performed using inductively coupled plasma–emission spectrometry (ICP–OES). The measured Ru loading is low, with a value of 0.5 mg cm^−2^. This loading is even notably lower than the industrial–level IrO_2_ loading at 2–4 mg cm^−2^. In order to further determine the degree of Ru ion exchange, we calculated the Mn/Ru molar ratio. The Mn/Ru ratio is 0.037, indicating that the majority of Mn is replaced by Ru after the Ru ion exchange (Table S1). X-ray diffraction (XRD) is used to determine the structure information of α–MnO_2_ nanoarrays and Mn–RuO_2_ (300) nanoarrays. As presented in Figure S3, the patterns for both the α–MnO_2_ nanoarrays and Mn–RuO_2_ (300) nanoarrays present characteristic peaks corresponding to metallic Ti. No visible characteristic peaks for MnO_2_ or RuO_2_ are observed, which can be attributed to their low loading levels, as revealed by the ICP–OES results.

**Figure 2:**
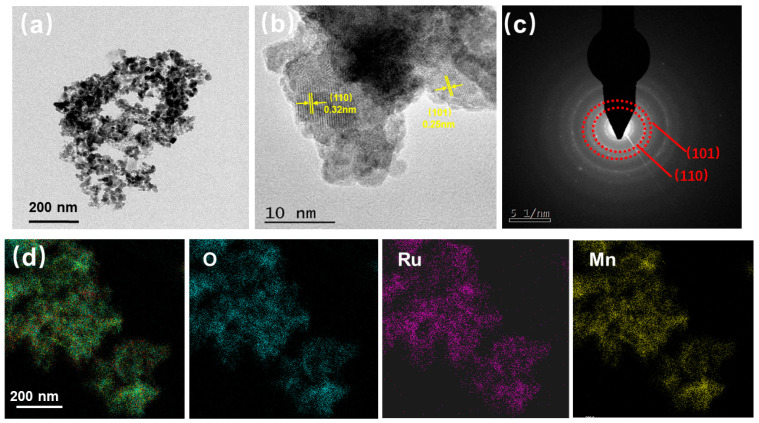
TEM images of Mn–RuO_2_ (300) nanoarrays (**a**,**b**) and the corresponding SAED pattern (**c**), STEM image (**d**), and elemental mapping images of O, Ru, and Mn.

To further investigate the structure and morphology features of Mn–RuO_2_ nanoarrays, high–resolution transmission electron microscopy (HRTEM) was employed. As depicted in Figure 2b, the particle size of Mn–RuO_2_ (300) is about 5 nm, which is different from α–MnO_2_, which possesses nanowire morphology (Figure S4). Distinct lattice fringes are observed, with the lattice spacing aligning with the crystallographic planes of rutile RuO_2_. A lattice spacing value of 0.32 nm can be ascribed to the (110) plane of rutile RuO_2_ and a lattice spacing value of 0.25 nm corresponds to the (101) plane of rutile RuO_2_. It is important to note that the crystal planes observed in the HRTEM images exhibit random orientations in various directions, signifying the polycrystalline nature of the Mn–RuO_2_ (300) nanoarrays. To further determine the crystal orientation of the Mn–RuO_2_ (300) nanoarrays, we employed a selected–area electron diffraction (SAED) measurement. Figure 2c illustrates the SAED pattern of the Mn–RuO_2_ (300) nanoarrays, which is consistent with the HRTEM analysis and can be indexed to the (110) and (101) planes of Mn–RuO_2_ (300) nanoarrays. Element mapping provides further confirmation of the chemical composition and distribution of the different elements within the Mn–RuO_2_ (300) nanoarrays, as displayed in Figure 2d, where a uniform distribution of Ru, Mn, and O elements is observed throughout the nanostructure.

**Figure 3:**
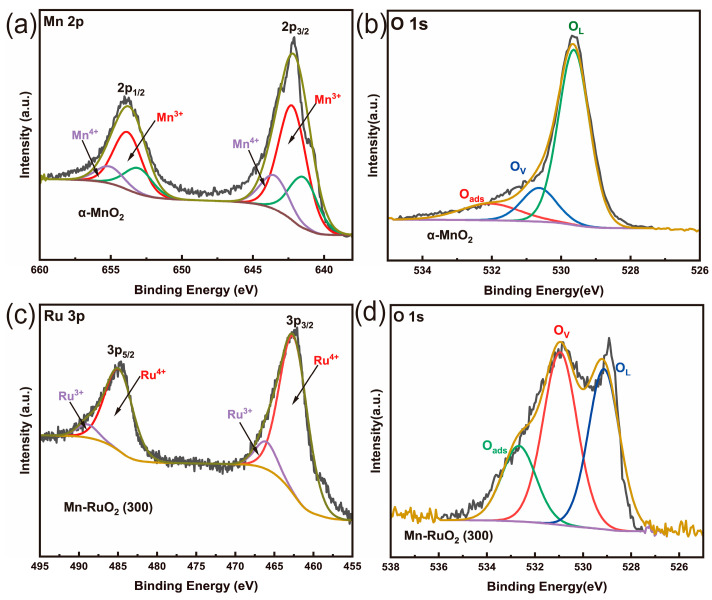
High–resolution Mn 2p and O 1s XPS spectra of α–MnO_2_ nanoarrays (**a**,**b**) and Ru 3p and O 1s XPS spectra of Mn–RuO_2_ nanoarrays (**c**,**d**).

To examine the surface chemical states of the α–MnO_2_ nanoarrays and Mn–RuO_2_ (300) nanoarrays, an X-ray photoelectron spectroscopy (XPS) analysis was conducted. Figure 3a illustrates the Mn 2p spectra of the α–MnO_2_ nanoarrays, revealing two sets of doublet peaks within the range of 640 eV to 660 eV. These doublet peaks correspond to the Mn^3+^ and Mn^4+^ 2p_1/2_ and 2p_3/2_ states. Specifically, the peaks at 641.22 eV and 653.05 eV are indicative of Mn^3+^, while the peaks at 644.22 eV and 653.82 eV are associated with Mn^4+^. Figure 3b illustrates the O 1s spectra of the α–MnO_2_ nanoarrays; three distinctive peaks are observed at 529.44 eV, 531.34 eV, and 532.84 eV, representing lattice oxygen, oxygen vacancies, and surface–adsorbed water molecules, respectively [22,23,24]. It is noteworthy that the predominant oxygen species is lattice oxygen. This observation signifies the favorable crystallinity of α–MnO_2_. Turning to the O 1s spectrum of the Mn–RuO_2_ (300) nanoarrays (Figure 3d), we observe that the content of lattice oxygen decreases from 71.35% to 31.68%, while the content of oxygen vacancies increases from 15.47% to 47.52% compared to the α–MnO_2_ nanoarrays. This result can be ascribed to more defects of the α–MnO_2_ nanoarrays after the Ru ion exchange. Furthermore, there is an augmentation in the proportion of surface–adsorbed water molecules from 13.19% to 20.30%. This heightened capability of the Mn–RuO_2_ (300) nanoarrays to accommodate surface–adsorbed water molecules potentially enhances the efficacy of the oxygen evolution reaction (OER) process. As the XPS spectrum of the Ru 3d peak partially overlaps with the C 1s region, the oxidation state of Ru is commonly discerned based on its binding energy values in the Ru 3p region [25,26]. Despite the variation in sensitivity between the 3d and 3p levels, the discrepancy is not significant enough to impede data acquisition or hinder the utilization of the 3p orbitals for both quantitative and qualitative analyses, as indicated by the relative sensitivity factors provided by Wagner [27]. As shown in Figure 3c, the Mn–RuO_2_ (300) nanoarrays exhibit a Ru 3p_3/2_ peak, consistent with the observable Ru 3p_3/2_ peaks for RuO_2_. The Ru 3p_3/2_ spectra of the Mn–RuO*_2_* (300) nanoarrays are situated at 462.52 eV and 466.0 eV, corresponding to Ru^4+^ and Ru^3+^ states, respectively [28]. It is obvious that the content of Ru^4+^ is predominant in the Mn–RuO*_2_* (300) nanoarrays.

**Figure 4:**
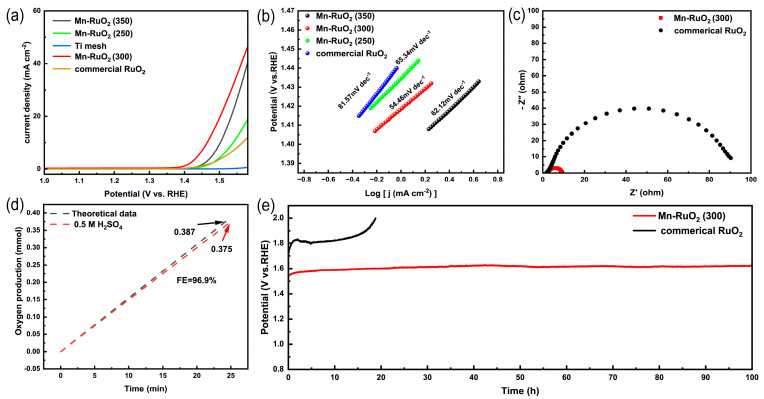
LSV curves for Mn–RuO_2_ nanoarrays, Ti mesh, and commercial RuO_2_ (**a**); the corresponding Tafel plots (**b**) and EIS curves (**c**); the FE plot of Mn–RuO_2_ (300) nanoarrays (**d**); and the chronopotentiometry test of Mn–RuO_2_ (300) nanoarrays and commercial RuO_2_ at 10 mA cm^−2^ in 0.5 M H_2_SO_4_ (**e**).

To evaluate the performance of the catalysts for the OER in acidic solutions (0.5 M H_2_SO_4_), an electrochemical three–electrode system was utilized. Figure 4a presents the linear sweep voltammetry (LSV) curves of the Mn–RuO_2_ nanoarrays, Ti mesh, and commercial RuO_2_. Negligible OER activity is observed on the Ti mesh, reaffirming its role solely as a current collector. Among the Mn–RuO_2_ nanoarray samples, the Mn–RuO_2_ (300) nanoarrays demonstrate optimized acidic OER activity, displaying a low overpotential of 217 mV at 10 mA cm^−2^, which is significantly lower than that of commercial RuO_2_ (320 mV at 10 mA cm^−2^). The OER activity of the Mn–RuO_2_ (300) nanoarrays also aligns with that of the reported Ru–based electrocatalysts, with exceptional performance (Table S3). Analysis of the Tafel slope in Figure 4b reveals that the Mn–RuO_2_ (250) nanoarrays, Mn–RuO_2_ (300) nanoarrays, and Mn–RuO_2_ (350) nanoarrays exhibit similar values. The Tafel slope value of Mn–RuO_2_ (250), Mn–RuO_2_ (300), and Mn–RuO_2_ (350) are, respectively, 65.34 mV dec^−1^, 54.46 mV dec^−1^, and 62.12 mV dec^−1^. All of the above values are significantly smaller than commercial RuO_2_ (81.57 mV dec^−1^), signifying the higher efficiency of the OER process. Reasonably, a lower annealing temperature, such as 250 °C, results in poor transformation of Ru^3+^ to Ru^4+^, as well as poor formation of RuO_2_. A higher annealing temperature would disrupt the integrity of the nanoarrays, leading to poor activity. The Tafel slope of the Mn–RuO_2_ (300) nanoarrays is also comparable to those of the excellent Ru–based electrocatalysts previously reported [10]. Electrochemical impedance spectroscopy (EIS) was utilized to investigate the dynamic characteristics of the electrode–electrolyte interface during OERs. The semicircle in the high–frequency region represents the charge transfer resistance (R_ct_), which directly correlates with electrocatalytic performance. Smaller R_ct_ values indicate higher electron transfer rates. As depicted in Figure 4c, the high–frequency semicircle of the Mn–RuO_2_ (300) nanoarrays is substantially smaller than that of RuO_2_, confirming faster electron transfer and higher OER activity. The interconnected nanoarrays provide the high exposure of catalytic sites and intensively void spaces which can greatly facilitate electron/mass transfer and gas desorption, thus accelerating the reaction process. Additionally, the Mn dopant can modify the electronic structure of Ru sites, enhancing the intrinsic activity of Ru [29,30,31]. Furthermore, we compared the electrochemical active surface area (ECSA) of both Mn–RuO_2_ (300) nanoarrays and commercial RuO_2_ by a double–layer capacitance (C_dl_) method. As illustrated in Figure S5, the Mn–RuO_2_ (300) nanoarrays presented a C_dl_ value of 7.91 mF cm^−2^, while the commercial RuO_2_ merely exhibited a C_dl_ value of 0.85 mF cm^−2^, indicating that the Mn–RuO_2_ (300) nanoarrays could expose a greater number of active sites, corresponding to the EIS analysis. The stability of the Mn–RuO_2_ (300) nanoarrays was assessed through chronopotentiometry testing. As illustrated in Figure 4e, the Mn–RuO_2_ (300) nanoarrays maintain OER activity at 10 mA cm^−2^ for over 100 h, with a negligible increase in overpotential. Conversely, the overpotential of the commercial RuO_2_ experiences a rapid escalation in less than 20 h. Furthermore, the oxygen Faradaic efficiency (FE_oxygen_) of the Mn–RuO_2_ (300) nanoarray’s catalyst was calculated to determine the utilization for O_2_ production. As shown in Figure 4d, the value of FE_oxygen_ is about 96.9%, indicating that the applied current could be efficiently utilized for O_2_ production. In addition, the stability number (S–number), an independent metric for electrocatalyst stability assessment which is unaffected by catalyst loading, accessible surface area, or the catalytic sites involved, was employed for stability evaluation. The S–number, defined as the ratio of the amount of evolved oxygen to the amount of dissolved catalysts, was calculated by measuring the Ru dissolved during the 100 h chronoamperometry measurement at a current density of 10 mA cm^−2^ in an acidic solution containing 0.5 M H_2_SO_4_ via ICP–OES. The calculated S–number is 2.7 × 10^4^, which is even close to the values of some Ir–based electrocatalysts [32,33]. The high value of the S–number indicates that the Mn–RuO_2_ (300) nanoarray’s catalyst possesses excellent corrosion resistance in an acidic environment. The remarkable OER performance of the Mn–RuO_2_ (300) nanoarrays can be attributed to the high exposure of active sites, efficient electron and mass transfer, as well as the electronic modification by Mn doping.

**Figure 5:**
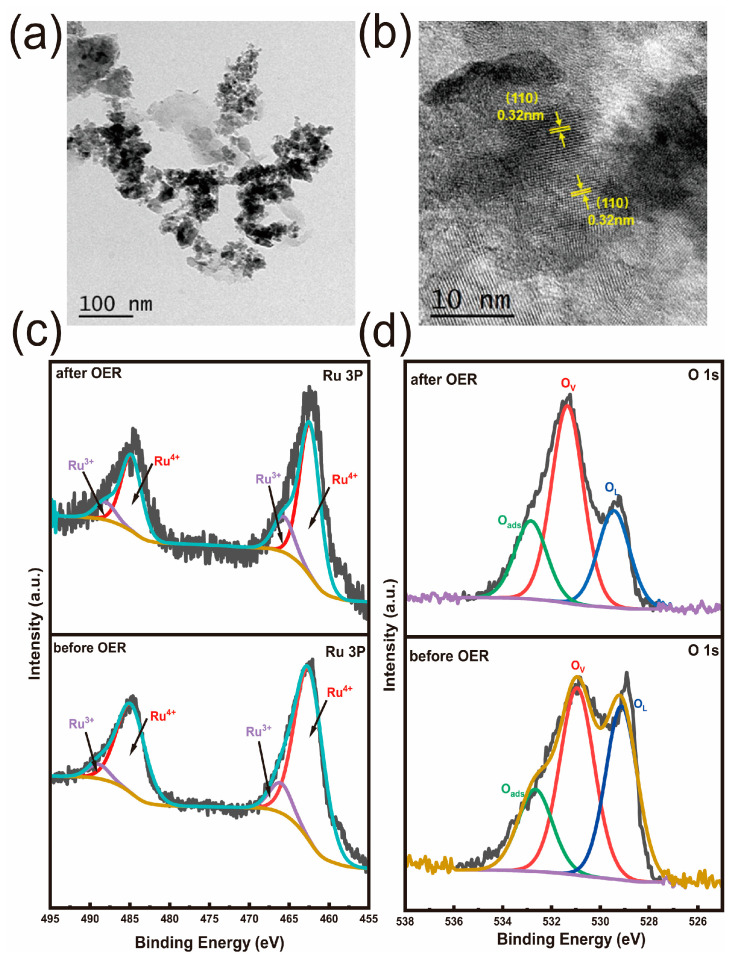
TEM images of Mn–RuO_2_ (300) nanoarrays after chronoamperometry test (**a**,**b**) and Ru 3p O 1s XPS spectra of Mn–RuO_2_ (300) nanoarrays before and after chronoamperometry test (**c**,**d**).

To obtain morphology and structure information of the Mn–RuO_2_ (300) nanoarrays, which experienced the 100 h chronopotentiometry test at 100 mA cm^−2^ in an acidic solution containing 0.5 M H_2_SO_4_, we employed SEM, TEM, and XPS characterizations. The morphology of the Mn–RuO_2_ (300) nanoarrays after the 100 h chronopotentiometry test is generally maintained (Figure S6). The TEM and HRTEM images (Figure 5a,b) reveal that the Mn–RuO_2_ (300) nanoarrays generally maintains their initial nanoparticle morphology and crystal structure after undergoing the chronoamperometry test. The uniform distribution of the Mn element from the element mappings after chronopotentiometry demonstrate the excellent stability of the Mn–RuO_2_ (300) nanoarrays (Figure S7). The XPS analysis of the Mn–RuO_2_ (300) nanoarrays before and after the chronoamperometry test is depicted in Figure 5c,d. After the chronoamperometry test, the Ru 3p binding energy experiences little shift, suggesting no significant valence changes in Ru after the chronoamperometry test (Figure 5c). The O 1s spectrum exhibits three peaks at 529.13 eV, 530.95 eV, and 532.66 eV (Figure 5d) [34], corresponding to lattice oxygen, oxygen vacancies, and surface–adsorbed oxygenous species, respectively. Upon the stability test, only the proportion of lattice oxygen in the Mn–RuO_2_ (300) nanoarrays decreased, which was due to surface amorphization of the Mn–RuO_2_ (300) nanoarrays [35,36]. To ascertain whether the Mn–RuO_2_ (300) nanoarrays will undergo reconstruction during the OER test, we induced an in situ Raman analysis. As depicted in Figure S8, the two distinct peaks at 526 cm^−1^ and 633 cm^−1^ can be ascribed to the signals originating from the Ru–O bonds. Notably, no additional peaks emerged in the Raman spectrum throughout the entire potential range from 1.133 V to 1.433 V. This observation strongly indicates the inherent stability of the Mn–RuO_2_ (300) nanoarray’s structure.

## 3. Materials and Methods

### 3.1. Materials

Potassium permanganate (KMnO_4_), ruthenium trichloride (RuCl_3_), ruthenium oxide (RuO_2_), acetone (C_3_H_6_O), absolute ethanol (C_2_H_6_O), and Nafion solution (5 wt% in a mixture of lower aliphatic alcohols and water, containing 45% water) were procured from Sinopharm Chemical Reagent Co., Ltd., (Ningbo, China). Deionized water (DI H_2_O) was prepared in–house, while titanium (Ti) meshes were obtained from Hebei Chaochuang Metal Mesh Industry (Hengshui, China). The counter electrode (platinum mesh), work electrode holder, reference electrode (Hg/Hg_2_SO_4_), and carbon paper were purchased from Gaoshi Ruilian (Tianjin, China) Photoelectric Technology Co., Ltd., (Tianjin, China). Prior to usage, the Ti meshes underwent a 30 min ultrasonic treatment involving acetone, absolute ethanol, and deionized water.

### 3.2. Preparation of Mn–RuO_2_ Self–Supporting Electrode

All the chemical reagents utilized in this experiment were of analytical purity and were used without any further purification. A straightforward hydrothermal method was employed to synthesize manganese dioxide (MnO_2_) nanoarrays on a Ti mesh. The Ti mesh was positioned within a 100 mL container made of polytetrafluoroethylene, with its inner wall filled with 35 mL solution containing 54.95 mg KMnO_4_. Afterward, the mesh was transferred to a high–temperature reactor fabricated from stainless steel. A 4 h reaction was carried out at 160 °C to synthesize a nanoarray of α–MnO_2_ on the mesh. Following the reaction, the mesh was rinsed with water and left to naturally cool down before being dried at 70 °C. Afterwards, the dried Ti mesh underwent immersion in a pre–prepared solution of RuCl_3_, which consisted of 10 mg of RuCl_3_ dissolved in 10 mL ethanol, and was stirred in darkness at room temperature for 12 h. The mesh was then rinsed with deionized water and dried at 70 °C in the absence of light. The resulting product was subsequently annealed at 300 °C for 2 h in air and designated as Mn–RuO_2_ (300). The synthesis procedures for Mn–RuO_2_ (250) and Mn–RuO_2_ (350) remained identical to the aforementioned process, with the exception being that the annealing temperatures were adjusted to 250 °C and 350 °C, respectively. For comparative purposes, electrochemical testing of commercially available RuO_2_ was conducted using the following method: 4 mg of RuO_2_ was dispersed in a solution consisting of 980 μL of isopropanol, 1 mL of ethanol, and 20 μL of Nafion solution to formulate an ink solution. The ink solution was subjected to 30 min of ultrasonic treatment, after which it was deposited onto a 1 cm × 1 cm carbon paper substrate.

### 3.3. Characterization

Scanning images were taken using scanning electron microscopy (SEM, Hitachi S–4800, Hitachi, Tokyo, Japan). The sample morphologies and structures were examined using Transmission Electron Microscopy (TEM, Tecnai F20, JEM–ARM200F, JEOL Ltd., Tokyo, Japan). The sample was prepared as follows. Firstly, the Mn–RuO_2_ (350) nanoarrays on Ti mesh were immersed in a glass bottle containing 10 mL alcohol. Secondly, the glass bottle was subjected to 30 min of ultrasonic treatment. Finally, 50 μL solution in the glass bottle was deposited onto a copper microsphere. The high–resolution images were captured at high voltages, and the elements’ distributions were analyzed with an Energy Dispersive Spectrometer (EDS). X-ray powder diffraction (XRD) analysis was conducted using a D8–Advance Davinci diffractometer with Cukα (λ = 1.5418 Å) radiation at room temperature. Surface elemental components and chemical states of the samples were analyzed using X-ray Photoelectron Spectroscopy (XPS, Axis SUPRA, Kratos, Manchester, UK). The elemental content of the samples was analyzed using inductively coupled plasma–emission spectrometry (ICP–OES, Spectro Arcos, SPECTRO Analytical Instruments, Inc., Mahwah, NJ, USA). The sample preparation process was as follows: Initially, the weight of the Mn–RuO_2_ (350) nanoarrays on Ti mesh sample was measured, yielding a weight of 25.5 mg. Subsequently, the sample was transferred into a high–temperature and high–pressure reactor, where 2 mL hydrofluoric acid (HF), 2 mL hydrochloric acid (HCl), and 4 mL nitric acid (HNO_3_) was sequentially added. Then, the reaction was conducted at 180 °C for 4 h. Following natural cooling, the resulting transparent solution was transferred to a 100 mL volumetric flask, and the volume was adjusted with water up to the mark. The preparation of the standard solution involved the following steps: Four volumetric flasks with capacities of 50 mL,100 mL, 50 mL, and 25 mL were chosen and designated as S0–S3. Subsequently, another 10 mL volumetric flask was taken and 10 mg of Mn standard solution was introduced, and then it was filled with water up to the mark. Using a pipette, 100 μL was dispensed into each of the S1–S3 volumetric flasks in the subsequent step. Then, 20 μL of Ru standard solution was taken and added to each of the S1–S3 volumetric flasks. Lastly, 1 mL, 2 mL, 1 mL, and 0.5 mL of nitric acid was added to the S0–S3 volumetric flasks and they were filled with water up to the mark. In situ Raman tests were performed using a Ren–ishaw inVia Reflex Raman spectrometer in a homemade three–electrode cell containing 0.5 M H_2_SO_4_ electrolyte. Mn–RuO_2_ (300) nanoarrays on Ti mesh, Ag/AgCl, and Pt wire were utilized as the working electrode, reference electrode, and counter electrode, respectively. The potential was incrementally raised from 1.133 V to 1.433 V, and the resulting Raman signals were captured. 

### 3.4. Electrochemical Measurements

The electrochemical measurements were conducted using a CHI760E electrochemical workstation (Shanghai Chenhua, Shanghai, China) in a three–electrode system. The counter electrode consisted of a platinum mesh (1 cm × 1 cm), while the reference electrode was a Hg/Hg_2_SO_4_ electrode. All experiments were performed at room temperature in a 0.5 M H_2_SO_4_ electrolyte. Cyclic voltammograms (CVs) were recorded with a scan rate of 50 mV s^−1^ with a potential range from 1.0 V to 1.6 V vs. RHE. Linear sweep voltammetry (LSV) tests were conducted at a scan rate of 5 mV s^−1^ with a potential range from 1.0 V to 1.6 V vs. RHE. Prior to conducting the LSV test, the sample underwent 100 cycles of CV until repetitive curves were achieved. The chronopotentiometry test was carried out at 10 mA cm^−2^ in an acidic solution containing 0.5 M H_2_SO_4_ using a two–electrode system. Electrochemical impedance spectra (EIS) were measured from 1000 kHz to 0.01 kHz at 1.45 V vs. RHE with an amplitude of 5 mV. The electrochemical double–layer capacitance of Mn–RuO_2_ (300) nanoarrays and commercial RuO_2_ were measured by different scanning rate CVs from 120 mV s^−1^ to 20 mV s^−1^ with an interval 20 mV s^−1^. The potential range was from 0.96 V to 1.06 V. The Tafel slope was calculated by the Tafel formula of η = a + b × lg (I), where η represented the overpotential of OER, a represented the Tafel constant, b represented the Tafel slope, and I represented the absolute value of current density corresponding to the overpotential from LSV. The Faraday efficiency (FE) was calculated using the bubbling method, which involved recording the rising volume (V) of the soap bubble and the total number of charges transferred under a constant current of 100 mA cm^−2^. The calculation for FE was as follows: FE = n × F × V/(1000 × V_m_ × It), where n denoted the electron transfer number of OER (the value of n is 4 for OER), F represented the Faraday constant (96,485 C mol^−1^), V denoted the volume change in oxygen production (mL), and V_m_ represented the molar volume (24.5 L mol^−1^ under normal temperature and pressure), and it corresponded to the total charge transferred.

## 4. Conclusions

Through the annealing of Ru–exchanged α–MnO_2_ nanoarrays, we have successfully fabricated self–supported Mn–RuO_2_ (300) nanoarrays. Utilizing the advantages of the high exposure of active sites, efficient electron and mass transfer, as well as the electronic modification from the Mn dopant, the resulting Mn–RuO_2_ (300) demonstrates remarkable acidic OER activity and stability. Notably, the S–number of Mn–RuO_2_ (300) surpasses existing catalyst stability metrics, underscoring its exceptional stability in acidic OERs. This study highlights the considerable potential of self–supported metal–doped Ru oxides for acidic water electrolysis.

## Data Availability

Data are contained within the article and Supplementary Materials.

## Supplementary Materials

The following supporting information can be downloaded at: https://www.mdpi.com/article/10.3390/molecules28237727/s1. Figure S1: SEM of (a,c) Mn–RuO_2_ (250) and (b,d) Mn–RuO_2_ (350). Figure S2: SEM images of commercial RuO_2_ (a,b) and Mn–RuO_2_ (c,d). Figure S3: XRD pattern of Mn–RuO_2_ (300) and α–MnO2 nanoarrays. Figure S4: TEM images of α–MnO_2_. Figure S5: The double–layer capacitances of Mn–RuO_2_ (300) nanoarrays and commercial RuO_2_. Figure S6: SEM images of Mn–RuO_2_ (300) nanoarrays after 100 h chronopotentiometry test. Figure S7: STEM images and elemental mapping images of O, Ru, and Mn of Mn–RuO_2_ (300) nanoarrays after chronoamperometry test. Figure S8: In situ electrochemical Raman spectra of Mn–RuO_2_ (300) nanoarrays. Table S1: ICP–OES analysis of Mn–RuO_2_ (300). Table S2: ICP–OES analysis of dissolved Ru after stability test. Table S3: Comparison of OER activities between Mn–RuO_2_ (300) and other electrocatalysts in acidic solutions [37,38,39,40,41,42,43,44,45,46,47].
