# Supplementary material for: Self–Supporting Mn–RuO2 Nanoarrays for Stable Oxygen Evolution Reaction in Acid
Source: Molecules. 2023 Nov 23;28(23):7727. doi: 10.3390/molecules28237727 (PMC10708348; doi:10.3390/molecules28237727)
Supplement: Supplementary file 1 [file molecules-28-07727-s001.zip › molecules-2719720-supplementary.pdf]

Article

Self–Supporting Mn–RuO_2 Nanoarrays for Stable Oxygen Evolution Reaction in Acid

Mengting Deng [1,2,†], Yulong Tang [1,2,†], Zhiyi Lu [2,3,*], Yunan Wang [2,3,*] and Yichao Lin [2,3,*]

[1] School of Materials Science & Chemical Engineering, Ningbo University, Ningbo 315211, China
[2] Key Laboratory of Advanced Fuel Cells and Electrolyzers Technology of Zhejiang Province, Ningbo Institute of Materials Technology and Engineering, Chinese Academy of Sciences, Ningbo 315201, China
[3] School of Chemical Science, University of Chinese Academy of Sciences, Beijing 100049, China
* Correspondence: luzhiyi@nimte.ac.cn (Z.L.); wangyunan@nimte.ac.cn (Y.W.); yclin@nimte.ac.cn (Y.L.);
† These authors contributed equally to this work.

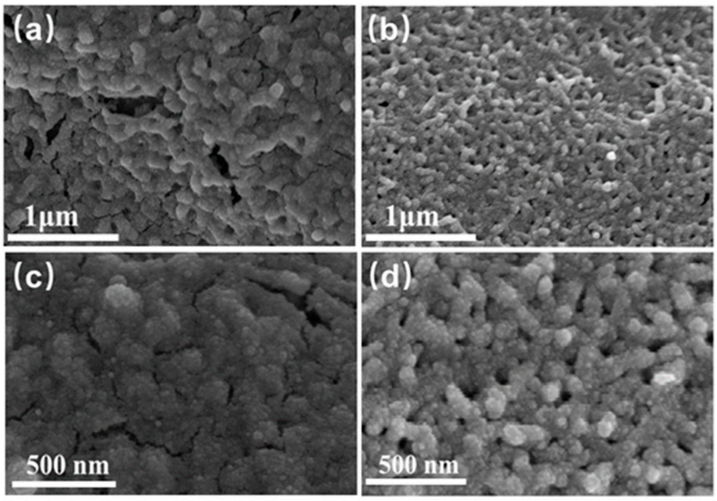

Figure S1: SEM of (a, c) Mn-RuO_2 (250) and (b, d) Mn-RuO_2 (350)

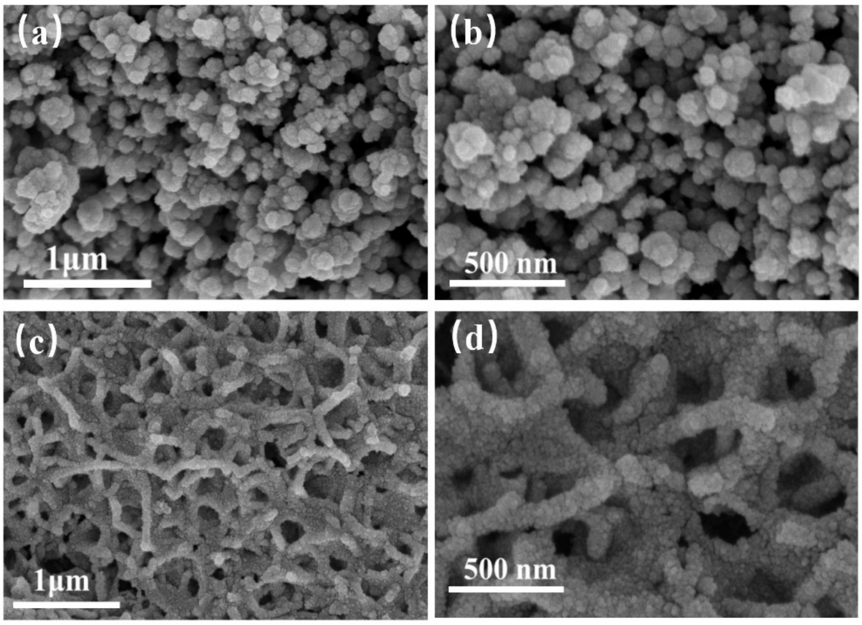

Figure S2. SEM images of commercial RuO_2 (a, b) and Mn-RuO_2 (300) (c, d).

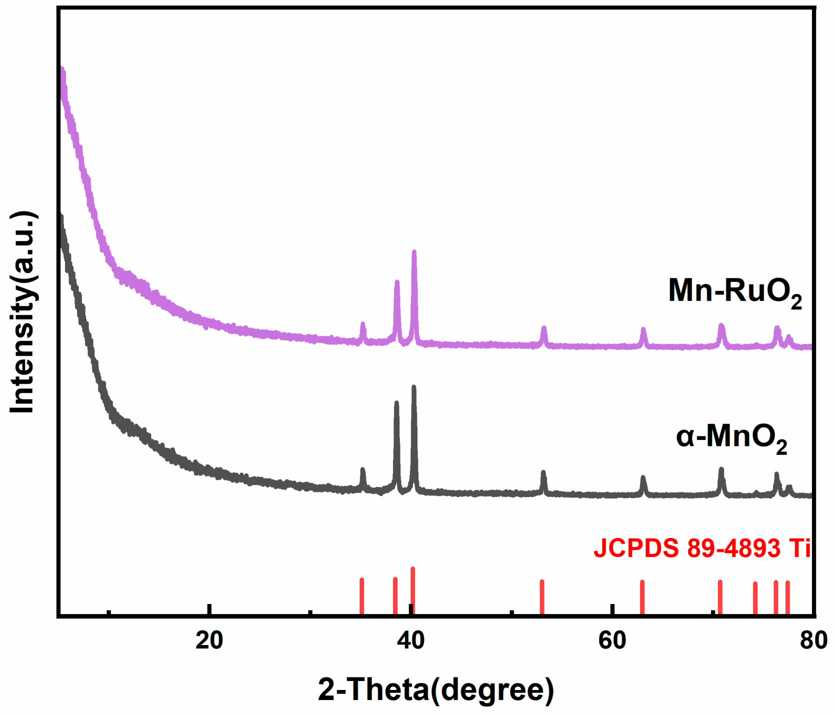

Figure S3. XRD pattern of Mn-RuO_2 (300) and α-MnO_2 nanoarrays.

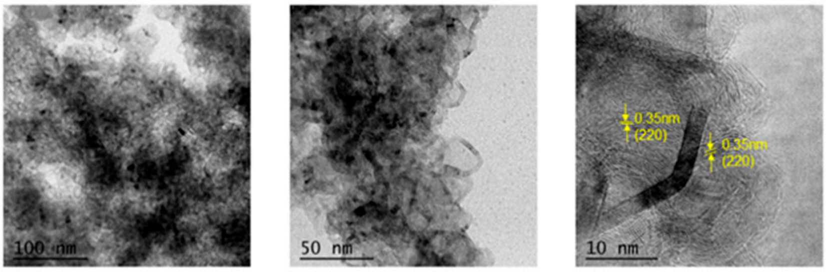

Figure S4: TEM images of α-MnO_2.

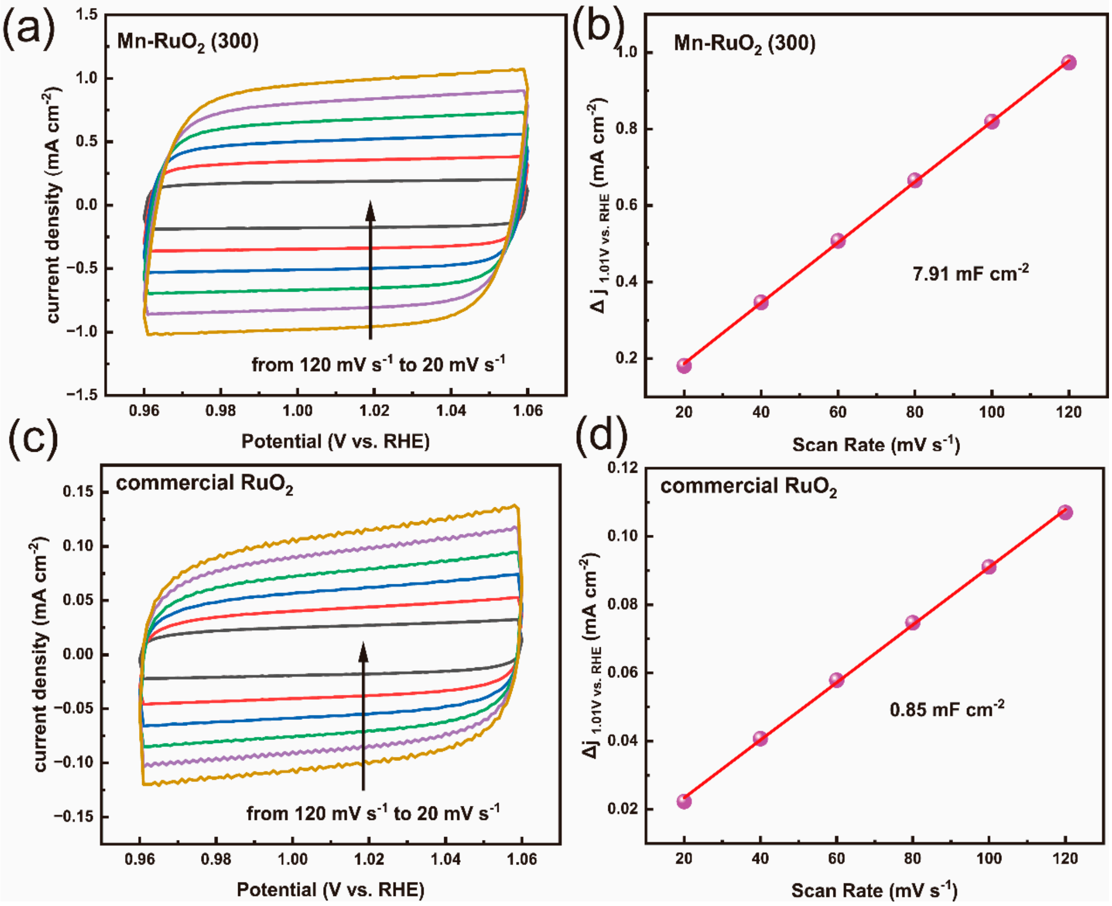

Figure S5. Measurements of the double layer capacitances. (a, b) Mn-RuO_2 (300) nanoarrays, (c, d) commercial RuO_2. All CV curves with scan rates ranging from 20 mV s^{-1} to 120 mV s^{-1} with an interval point of 20 mV s^{-1}.

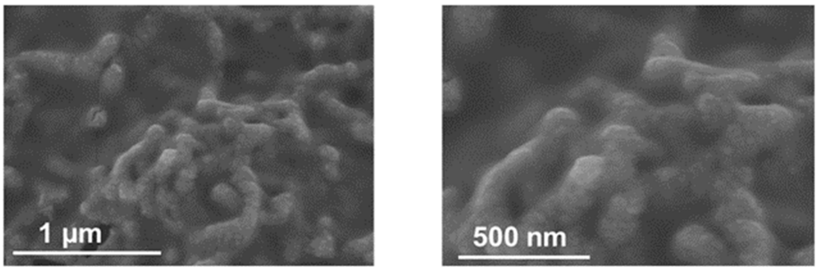

Figure S6: SEM images of Mn-RuO2 (300) nanoarrays after 100-h chronopotentiometry test.

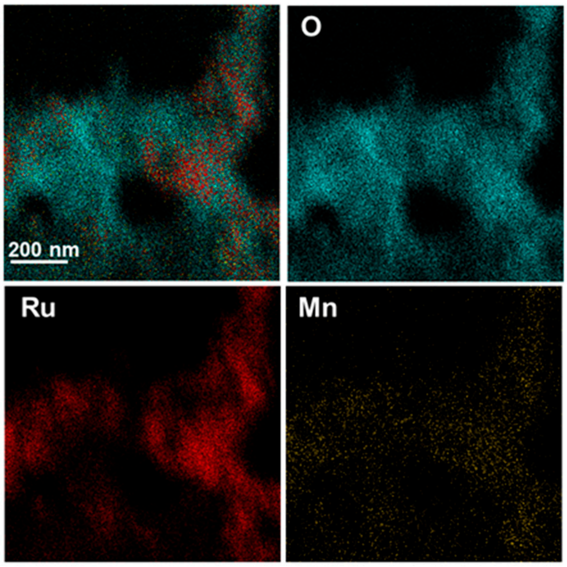

Figure S7: STEM images and elemental mapping images of O, Ru, and Mn of Mn-RuO_2 (300) nanoarrays after chronoamperometry test.

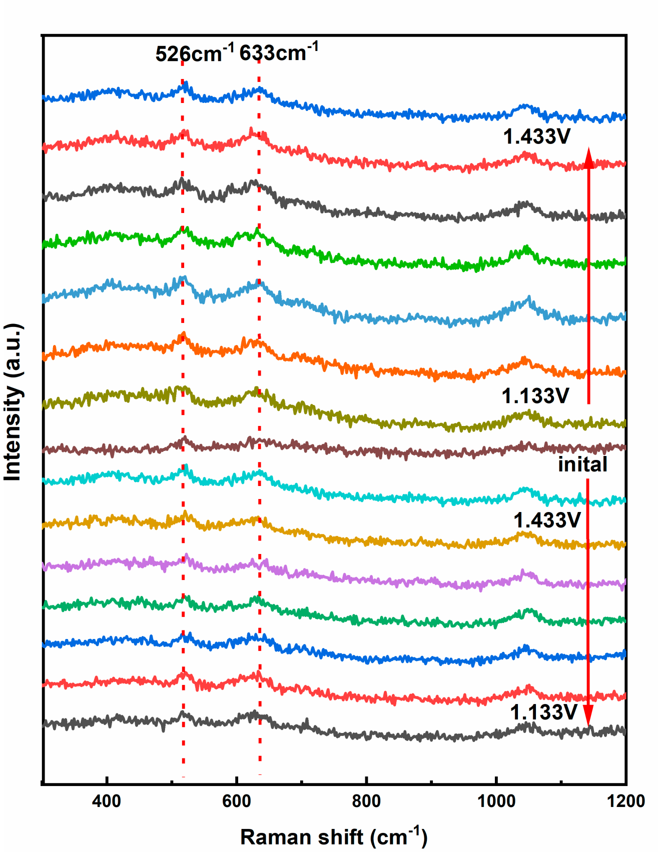

Figure S8. In-situ electrochemical Raman spectra of Mn-RuO_2 (300) nanoarrays.

Table S1: ICP-OES analysis of Mn-RuO_2 (300)

Sample	Ru (mg cm^{-2})	Mn (mg cm^{-2})
Mn-RuO_2 (300)	0.5	0.01

Table S2: ICP-OES analysis of dissolved Ru after stability test

Sample	Ru (mg)
Mn-RuO_2	0.0346

Table S3: Comparison of OER activities between Mn-RuO_2 (300) and other electrocatalysts in acidic solutions.

Electrocatalyst	J (mA·cm^{-2})	Overpotential(mV)	Stability	Electrolyte	Ref.
**Mn-RuO_2 (300)**	10	**217**	**100h**	0.5M H_2SO_4	**This work**
R20-Mn	10	210	20h	0.5M H_2SO_4	1
Mesoporous Ir nanosheets	10	240	8h	0.5M H_2SO_4	2
Mn–RuO_2	10	158	5000cycles	0.5M H_2SO_4	3
Ir–SA@Fe@NCNT	10	250	11.5h	0.5M H_2SO_4	4
Ru-N-C	10	267	30h	0.5M H_2SO_4	5
$Ir_{0.06}Co_{2.94}O_4$	10	297	200h	0.5M H_2SO_4	6
Ru@FLC	10	258	5000cycles	0.5M H_2SO_4	7
IrGa-IMC@IrO_x	10	272	3000cycles	0.1M $HClO_4$	8
IrRu@Te	10	220	20h	0.5M H_2SO_4	9
RuO_2/$(Co,Mn)_3O_4$/CC	10	270	24h	0.5M H_2SO_4	10
$(Ru, Mn)_2O_3$	10	168	40h	0.5M H_2SO_4	11

Reference

1. Zhao, Z. B.; Zhang, B.; Fan, D. Y.; Wang, Y. G.; Yang, H. J.; Huang, K.; Pan, X. C.; Zhang, R. M.; Tang, H. L.; Lei, M., Tailoring manganese oxide nanoplates enhances oxygen evolution catalysis in acid. J Catal. 2022, 406, 265-272.

2. Cang, B.; Guo, Y.; Kim, J.; Whitten, A. E.; Wood, K.; Kani, K.; Rowan, A. E.; Henzie, J.; Yamauchi, Y., Mesoporous Metallic Iridium Nanosheets. J. Am. Chem. Soc. 2018, 140, 12434-12441.

3. Chen, S.; Huang, H.; Jiang, P.; Yang, K.; Diao, J.; Gong, S.; Liu, S.; Huang, M.; Wang, H.; Chen, Q., Mn-Doped RuO_2 Nanocrystals as Highly Active Electrocatalysts for Enhanced Oxygen Evolution in Acidic Media. ACS Catal. 2020, 10, 1152-1160.

4. Luo, F; Hu, H.; Zhao, X.; Yang, Z.; Zhang, Q.; Xu, J.; Kaneko, T.; Yoshida, Y.; Zhu, C.; Cai, W., Robust and Stable Acidic Overall Water Splitting on Ir Single Atoms. Nano Lett. Nano Let. 2020, 20, 2120-2128.

5. Cao, L. L.; Luo, Q. Q.; Chen, J. J.; Wang, L.; Lin, Y.; Wang, H. J.; Liu, X. K.; Shen, X. Y.; Zhang, W.; Liu, W.; Qi, Z. M.; Jiang, Z.; Yang, J. L.; Yao, T., Dynamic oxygen adsorption on single-atomic Ruthenium catalyst with high performance for acidic oxygen evolution reaction. Nat. Commun. 2019, 10, 4849-4858.

6. Shan, J.; Ye, C.; Chen, S.; Sun, T.; Jiao, Y.; Liu, L.; Zhu, C.; Song, L.; Han, Y.; Jaroniec, M.; Zhu, Y.; Zheng, Y.; Qiao, S.-Z., Short-Range ordered iridium single atoms integrated into cobalt oxide spinel structure for highly efficient electrocatalytic water oxidation. J. Am. Chem. Soc. 2021, 143, 5201-5211.

7. Shi, C. X.; Yuan, Y.; Shen, Q.; Yang, X. D.; Cao, B. Q.; Xu, B.; Kang, B. T.; Sun, Y. Q.; Li, C. C., Encapsulated ruthenium nanoparticles activated few-layer carbon frameworks as high robust oxygen evolution electrocatalysts in acidic media. J. Colloid Interface Sci. 2022, 612, 488-495.

8. Chen, L. W.; He, F. X.; Shao, R. Y.; Yan, Q. Q.; Yin, P.; Zeng, W. J.; Zuo, M.; He, L. X.; Liang, H. W., Intermetallic IrGa-IrO_x core-shell electrocatalysts for oxygen evolution. Nano Res. 2022, 15 (3), 1853-1860.

9. Xu, J.; Lian, Z.; Wei, B.; Li, Y.; Bondarchuk, O.; Zhang, N.; Yu, Z.; Araujo, A.; Amorim, I.; Wang, Z.; Li, B.; Liu, L., Strong Electronic Coupling between Ultrafine Iridium-Ruthenium Nanoclusters and Conductive, Acid-Stable Tellurium Nanoparticle Support for Efficient and Durable Oxygen Evolution in Acidic and Neutral Media. ACS Catal. 2020, 10, 3571-3579.

10. Niu, S. Q.; Kong, X. P.; Li, S. W.; Zhang, Y. Y.; Wu, J.; Zhao, W. W.; Xu, P., Low Ru loading RuO_2/(Co,Mn)$_{(3)}O_{x-4}$ nanocomposite with modulated electronic structure for efficient oxygen evolution reaction in acid. Appl. Catal. B. 2021, 297, 926-934.

11.Qin, Y.; Cao, B.; Zhou, X.-Y.; Xiao, Z.; Zhou, H.; Zhao, Z.; Weng, Y.; Lv, J.; Liu, Y.; He, Y.-B.; Kang, F.; Li, K.; Zhang, T.-Y., Orthorhombic (Ru, Mn)$_2O_3$: A superior electrocatalyst for acidic oxygen evolution reaction. *Nano Energy.* **2023,** *115*, 108727.
